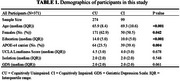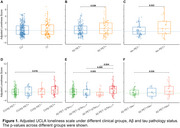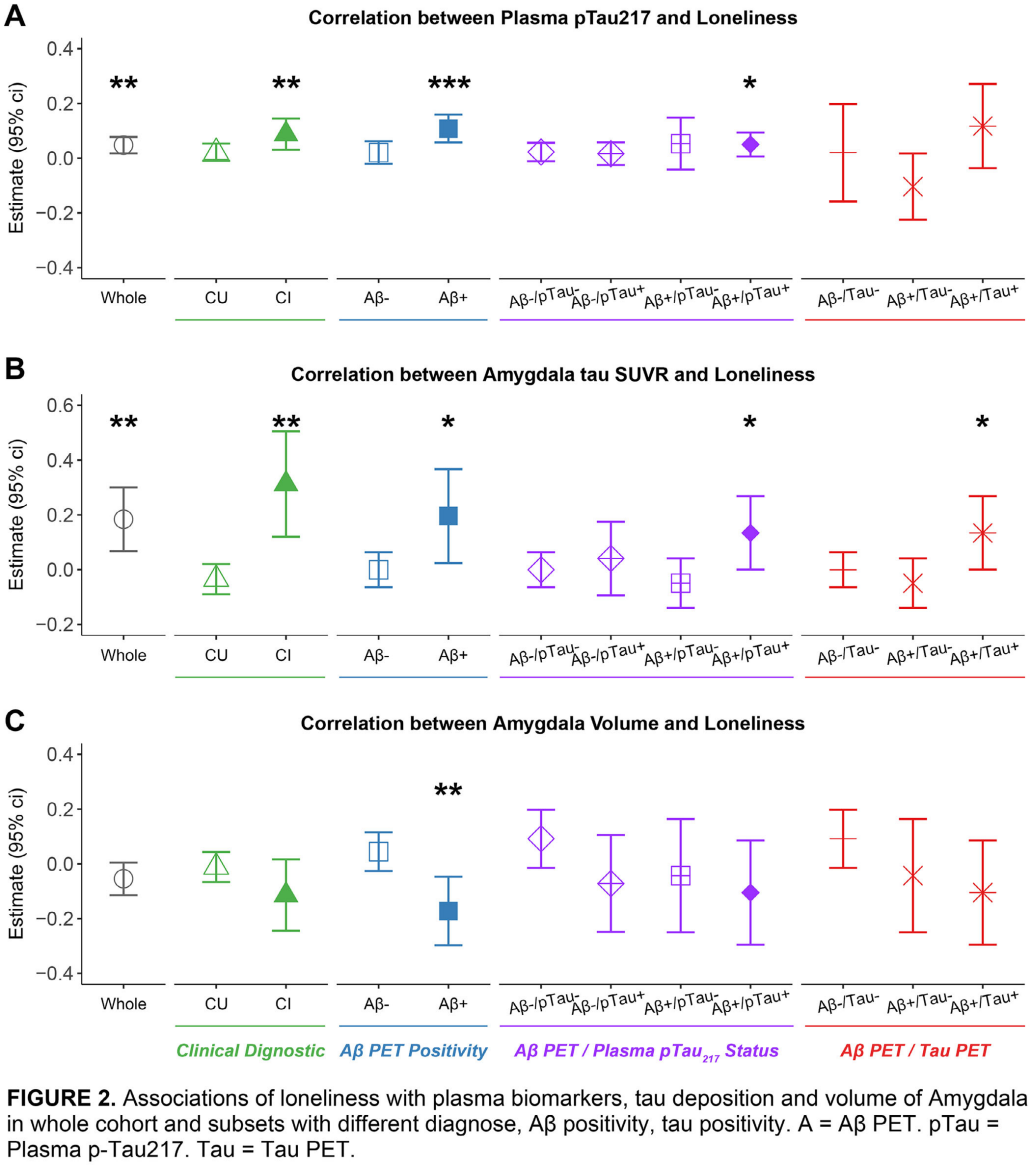# Lonely individuals show more tau pathology in Alzheimer's Disease

**DOI:** 10.1002/alz70857_100833

**Published:** 2025-12-25

**Authors:** Anqi Li, Lizhi Guo, Xin Zhou, Yiying Wang, Tengfei Guo

**Affiliations:** ^1^ Shenzhen Bay Laboratory, Shenzhen, Guangdong, China; ^2^ The Chinese University of Hong Kong, Hong Kong, Hongkong SAR, China; ^3^ Hainan University, Haikou, Hainan, China; ^4^ Peking University Shenzhen Graduate School, Shenzhen, Guangdong, China

## Abstract

**Background:**

Loneliness was associated with increased dementia risk and severity of Alzheimer's disease (AD) pathology, such as amyloid‐β (Aβ) and tau accumulation in the early stage. We aimed to assess how loneliness affects the process of AD pathology. Specifically, we focus on the amygdala, a brain region closely associated with loneliness.

**Method:**

274 cognitively unimpaired (CU) and 99 cognitively impaired (CI) participants from the Greater‐Bay‐Area Healthy Aging Brain Study (GHABS) were assessed for loneliness, isolation, and depression state by neuropsychiatric assessments. Among them, 274, 191, and 78 participants completed plasma phosphorylated tau_217_ (*p*‐Tau_217_), Aβ positron emission tomography (PET) scan, tau PET scan. The demographic is shown in Table 1. In separate linear regression models, we compared the UCLA‐loneliness score across different clinical groups (CU/CI), Aβ PET status (Aβ PET±), tau PET status (tau PET±), and plasma pTau_217_ status (pTau_217_±) by adjusting for social isolation, depression, education, age and sex. We subsequently evaluated the association of loneliness with plasma *p*‐Tau_217_, amygdala tau PET standard uptake value ratio (SUVR), and amygdala volume in the whole cohort, and subsets with different diagnosis (CU/CI), Aβ positivity (A±), tau positivity (T±).

**Result:**

Loneliness level showed no significant difference between diagnostic groups, while it rises with onset of AD pathology, especially with the onset of tau pathology (tau PET and plasma *p*‐Tau_217_) (Figure 1). Loneliness positively related to plasma *p*‐Tau_217_ and amygdala tau deposition in whole cohort, especially in CI, Aβ+ group or Aβ+/p‐Tau_217_+ (Figure 2). Amygdala volume showed a negative correlation with loneliness in the Aβ+ group.

**Conclusion:**

We found that the high loneliness level was associated with more tau pathology, especially in participants with cognitive impairment or who have Aβ pathology. These findings suggest a strong link between loneliness and tau pathology, providing novel insights into how the neuropsychiatry state affects tau aggregation in AD.